# A shot through the heart: a case report on retained bullet causing cardiac tamponade

**DOI:** 10.1093/ehjcr/ytae651

**Published:** 2024-12-06

**Authors:** Rakesh Shah, Thomas Dacey, Joseph Sebastian, Kenton Zehr, Shaun Cardozo

**Affiliations:** Department of Internal Medicine, DMC/Sinai Grace Hospital, 6071 Outer Dr W, Detroit, MI 48235, USA; Department of Emergency Medicine, DMC/Sinai Grace Hospital, 6071 Outer Dr W, Detroit, MI 48235, USA; Department of Cardiology, DMC/Harper University Hospital, 3990 John R St, Detroit, MI 48201, USA; Department of CardioVascular and Thoracic Surgery, DMC/Harper University Hospital, 3990 John R St, Detroit, MI 48201, USA; Department of Cardiology, DMC/Harper University Hospital, 3990 John R St, Detroit, MI 48201, USA

**Keywords:** Gunshot injury, Pericarditis, Tamponade, Sternotomy, Case report

## Abstract

**Background:**

As a rare complication of penetrating chest trauma, one can occasionally find foreign bodies inside the pericardium. Even rarer is finding an intact bullet inside the pericardial cavity following the gunshot injury.

**Case summary:**

A 17-year-old male presented to the emergency department as a Level 1 trauma for multiple gunshot wounds. Upon arrival, the patient was tachycardic but normotensive. Physical exam was notable for several penetrating wounds to the chest and right clavicle. The initial chest X-ray demonstrated a metallic foreign body consistent with a bullet overlying the cardiac silhouette. Approximately 24 h into the hospital course, ST-segment elevation was noted on telemetry. An electrocardiogram demonstrated sinus tachycardia with diffuse ST-segment elevation in all leads, consistent with acute pericarditis. Over the following several hours, the patient gradually developed tamponade physiology, prompting a more emergent median sternotomy.

**Discussion:**

Although penetrating cardiac injury carries a high mortality rate, management of these patients and complications that may arise during their hospital course are rarely explained. The diagnosis of projectile chest trauma starts with history and physical examination. The primary diagnostic modalities are the X-ray, computed tomography scan of the chest, electrocardiogram, and echocardiogram. Management of a patient with cardiac gunshot depends largely on haemodynamic status. As in our case, a patient with haemodynamic instability is managed with emergency exploration and removal of the foreign body.

**Learning points**

- In a similar situation of a gunshot wound near the cardiac region, an immediate electrocardiogram and bedside echocardiogram must be performed to assess for evidence of cardiac contusion, pericardial effusion, and pericarditis.
- Bullet is a large foreign body and if left inside it has a significant potential for erosion into vital structures. Therefore, authors believe it should be removed irrespective of symptoms.

## Introduction

According to the Centers for Disease Control and Prevention, firearm injuries accounted for more than 48 000 deaths in 2022, especially in young adults, with more than 40% of those deaths due to homicides.^1^ Foreign bodies, specifically a bullet following a gunshot wound (GSW) in the pericardial cavity, are rare and are primarily associated with penetrating chest trauma.^2–4^ These penetrating traumas are one of the causes of pericardial inflammation that can be complicated with cardiac tamponade and haemodynamic compromise.^5^ If not diagnosed and treated on time, it might lead to increased morbidity and mortality. Here, we present the case of a 17-year-old male who suffered multiple GSWs, with one bullet inside the pericardial cavity, which led to pericardial inflammation and tamponade.

## Summary figure

**Figure:**
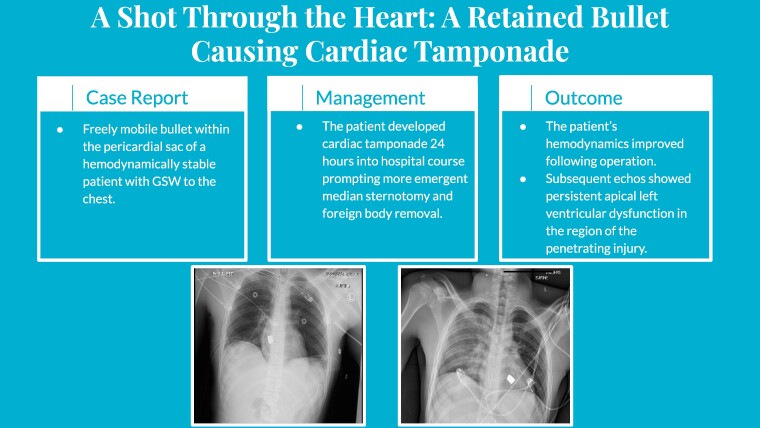


## Case presentation

A 17-year-old male presented to the emergency department as a Level 1 trauma for multiple GSWs. Upon arrival at the hospital, the patient was noted to be protecting his airway and had a Glasgow Coma Scale score of 14. Initial vital signs were significant for tachycardia and mildly elevated blood pressure. Patient was afebrile. The physical exam was notable for several penetrating wounds to the thorax, including in his left mandible, left upper chest, right clavicle, right lower chest, and lumbar region of the back over the midline. Cardiovascular examination was unremarkable for any abnormal heart sounds.

**Figure 1:**
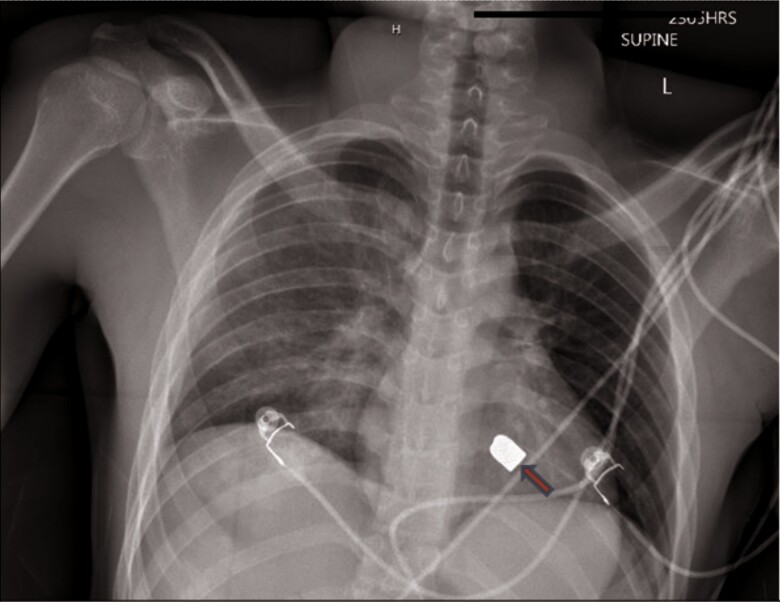
Chest X-ray at the time of arrival to the ED demonstrated a metallic foreign body consistent with a bullet overlying the cardiac silhouette.

An eFAST exam was positive for small pericardial effusion and a lack of lung sliding in the upper fields of the right lung. The initial chest X-ray demonstrated a metallic foreign body consistent with a bullet overlying the cardiac silhouette, in addition to a right-sided pulmonary contusion with no evident pneumothorax (*Figure 1*). The patient was transferred to the operating room for definitive management, where an exploratory laparotomy was performed and bilateral thoracostomy tubes were placed. The patient was started on ampicillin–sulbactam 3 g every 6 h. Pain was managed with opioids.

**Figure 2:**
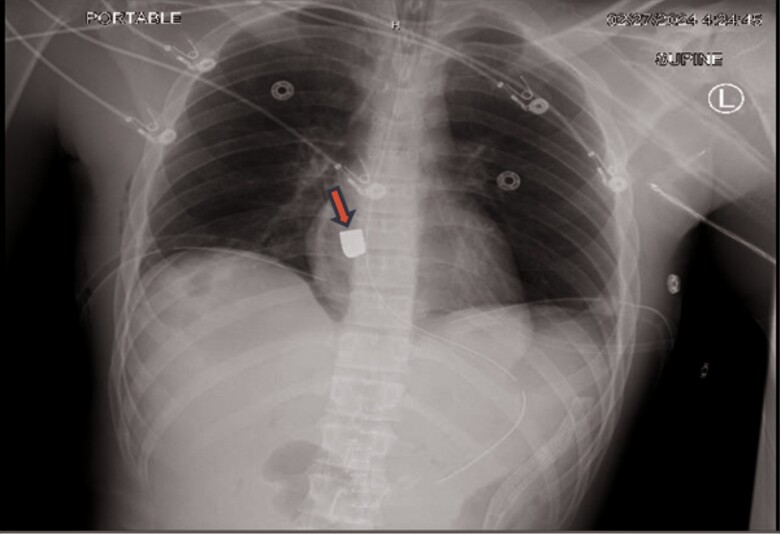
Repeat chest X-ray demonstrating a change in the bullet’s location and orientation.

Computed tomography (CT) thorax obtained post-operatively was notable for a moderate right-sided pneumothorax, contusion, and laceration in the upper and middle lobe of the right lung and post-surgical changes to intra-abdominal injuries. At that time, there was no evidence of pericardial effusion. The bullet was noted to be in the inferior aspect of the left hilar region. A chest X-ray (CXR) was repeated the following morning and was notable for migration and rotation of the bullet while still overlying the cardiac silhouette (*Figure 2*). On two chest X-rays separated by time, the foreign body had both moved and rotated, which suggested that it was within the pericardial sac and was freely mobile. Cardiothoracic surgery was consulted. As per the cardiothoracic surgery, there were no apparent signs of injury to vital structures; however, the bullet would require removal given the high potential of erosion into vital structures.

**Figure 3:**
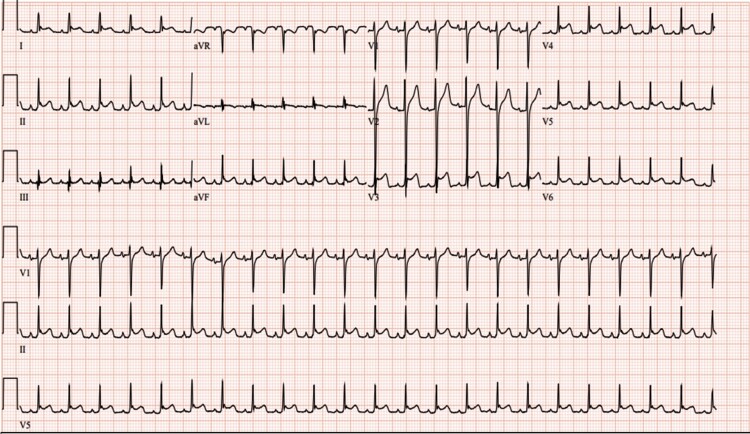
Electrocardiogram (EKG) demonstrating widespread concave ST-segment elevation and PR-segment depression with reciprocal ST-segment depression. Sinus tachycardia is also observed.

Approximately 24 h into the hospital course, ST-segment elevation was noted on telemetry. Patient did not have fever, and physical examination was unremarkable for diminished heart sounds or pericardial rub. A 12-lead electrocardiogram (ECG) demonstrated sinus tachycardia with diffuse ST-segment elevation in all leads (*Figure 3*). Troponin was markedly elevated at 18 198 ng/L. Cardiology was consulted, and likely explanation of above findings was thought to be pericardial inflammation secondary to injury due to the bullet. Patient did not receive any non-steroidal anti-inflammatory drugs up to this time. A transthoracic echocardiogram identified a small-moderate circumferential pericardial effusion with no haemodynamic evidence of cardiac tamponade or wall motion abnormalities. The pericardial effusion had foreshortened images of the left ventricular (LV) chamber, so the apical wall motion abnormality was not appreciated (Supplementary material online, Video S1). Over the following several hours, the patient gradually developed tamponade physiology, as suggested in the bedside echocardiogram. His vital signs demonstrated worsening tachycardia to the 130-s b.p.m. and gradually declining mean arterial pressures, prompting a more emergent median sternotomy.

**Figure 4:**
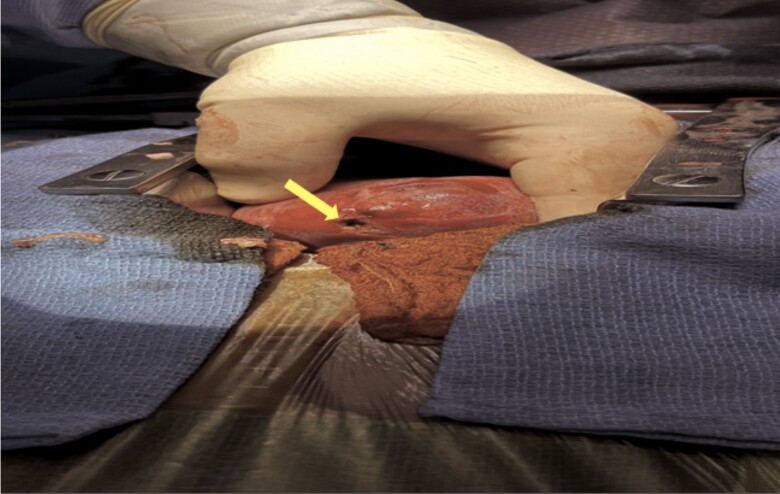
Intra-operative image of through-and-through injury to the apex of left ventricle.

According to the cardiothoracic surgery, the patient had a bullet that appeared to be external to vital structures but within his pericardial space. Because it appeared to be freely mobile within pericardial space, the best way to approach the pericardium was thought to be anterior. In this way, the heart and great vessels could be observed for any potential haematomas or injury, and the bullet can be easily located and removed. The median sternotomy incision is generally well tolerated, and the estimated mortality risk is extremely low with this approach. During the procedure, blood was evacuated from the pericardium. The bullet was identified in the posterior pericardium and was removed. Pericardial appeared inflamed, and through-and-through injury to the apex of the LV (*Figure 4*) was noted. There was no active bleeding from either side, the entry site appeared just to the left of the distal left anterior descending coronary artery, and the exit site was at the distal tip of the right ventricle. Both holes were repaired. An anterior mediastinal chest tube was placed, and the chest was closed.

The patient had a relatively uneventful post-operative course, with ECG changes and down-trending troponins resolved. Serial echocardiograms obtained following the surgical intervention showed resolution of pericardial effusion with apical LV dysfunction in the region of the through-and-through penetrating injury.

## Discussion

Pericarditis is one of the rare complications following a gunshot injury to the thorax or any other penetrating chest trauma. The onset of pericarditis following penetrating chest trauma can be immediate or delayed. In our case, the patient developed cardiac tamponade after 24–36 h following the injury. The authors believe that acute pericarditis was developing at the time of presentation and might have been unnoticed at the time of presentation as initial management was more focused on life-threatening abdominal injuries.

In a case report by Karmy-Jones *et al*.,^6^ the authors described two cases: a 39-year-old man who initially presented with a gunshot injury to the posterolateral left chest. He developed acute pericarditis after 8 days following the trauma. They also described a 25-year-old male who presented to the emergency department (ED) with a GSW to the xiphoid and underwent emergency laparotomy, which was complicated with pericardial tamponade on Day 13. In the study done by Valle^7^ in a group of chest casualties of the war victims who experienced thoracic trauma, 42 had pericardial effusion, primarily due to the presence of metallic fragments. Ten patients who were initially managed conservatively were readmitted with recurrent pericarditis between 4 and 26 months after the initial insult. In another case report, the author described a middle-aged male who presented to the ED with a GSW to the left chest. The patient was haemodynamically stable upon presentation. An echocardiogram showed no injury to the intra-cardiac structures and the absence of pericardial effusion. However, on the next day, the patient developed diffuse ST-segment elevations on electrocardiogram (EKG) consistent with acute pericarditis. Initially, he was asymptomatic and remained haemodynamically stable and was started on non-steroidal anti-inflammatory drugs, but on Day 3, bedside ultrasound showed a new moderate-sized pericardial effusion.^2^

The diagnosis of projectile chest trauma starts with history and physical examination. There may be obvious signs of physical injury to the chest. If present, CXR may show evidence of foreign bodies, along with haemothorax or pneumopericardium. CT of the chest can help to locate the accurate location of the foreign bodies along with other associated injuries to the thorax. An EKG can help to detect specific changes related to pericarditis, injury to coronary arteries, or disruption to the conduction system. An echocardiogram helps confirm pericardial tamponade in addition to providing information on structural damage to the heart.^8^

Management of a patient with cardiac gunshot depends largely on haemodynamic status. Post-traumatic pericarditis in a haemodynamically stable patient can be self-limiting, although recurrences are common. In the absence of tamponade, initial treatment consists of non-steroidal anti-inflammatory agents. As in our case, a patient with haemodynamic instability is managed with emergency exploration and removal of the foreign body. However, management remains challenging for asymptomatic patients. In a case report described by Mishra *et al*.,^4^ a 26-year-old male, following a gunshot injury to his chest, was admitted to the hospital. He was haemodynamically stable but was found to have a pellet at the posterior pericardial border in the vicinity of the inferior pulmonary vein, with no haemopericardium. An echocardiogram confirmed the presence of a retained bullet in the same position without any fluid collection and regional motion wall abnormalities. The patient was managed conservatively and was discharged. However, on Day 21, the patient had respiratory distress and eventually died, only later found to have cardiac tamponade secondary to massive haemorrhage of the pulmonary vein by the pellet.

In this context, some physicians recommend the removal of all intra-pericardial retained missiles irrespective of haemodynamic stability.^2^ Imaging, like a CT scan of the chest, is the most helpful tool in precisely locating the bullet within the pericardial cavity.^9^

The guidelines on these topics are not clear due to the rarity of the condition. The authors recommend an immediate EKG and bedside echocardiogram in a comparable situation of a GSW near the cardiac region to assess for evidence of cardiac contusion, pericardial effusion, and pericarditis. We recommend that bullets or any big foreign bodies inside or near the heart structure be removed, given the high potential of erosion into vital structures. The surgical approach can differ. For a freely mobile foreign body within pericardial space, like in our case, the best way to approach the pericardium was thought to be anterior. The median sternotomy incision has a low estimated mortality risk and is generally well tolerated among the patients.

## Conclusion

In a similar situation of a GSW near the cardiac region, an immediate EKG and bedside echocardiogram must be performed to assess for evidence of cardiac contusion, pericardial effusion, and pericarditis. Early surgical intervention in haemodynamically stable patients is paramount to prevent complications.

## Supplementary Material

ytae651_Supplementary_Data

## Data Availability

The data underlying this article will be shared on reasonable request to the corresponding author.

## Lead author biography

Rakesh Shah, MD, is an Internal Medicine resident at Wayne State University/Detroit Medical Center. He is interested in pursuing a career in cardiology with a keen interest in heart failure and cardiac imaging. Outside of medicine, he enjoys watching movies, travelling to new places, and hanging out with friends.

## Supplementary material

**Consent:** Consent was obtained from the patient regarding writing, publishing, and presenting this case. Authors endorse compliance with COPE guidelines.

**Funding:** None declared.
